# Frozen section evaluation of sentinel lymph nodes in breast carcinoma: a retrospective analysis

**DOI:** 10.3332/ecancer.2017.774

**Published:** 2017-10-18

**Authors:** Leonardo Russo, Luis Betancourt, Gabriel Romero, Alí Godoy, Laura Bergamo, Rafael Delgado, Ángela Ruiz, Marianna Gutiérrez, Eduardo Salas, Maria Puzzi

**Affiliations:** Dr Luis Razetti Oncology Institute, Caracas 1010, Venezuela

**Keywords:** Breast cancer, sentinel lymph node biopsy, frozen section, micrometastases

## Abstract

**Objective:**

To determine the false-negative rate, sensitivity, and diagnostic accuracy of the frozen section analysis of the sentinel lymph node (SLN) biopsy in early-stage breast cancer compared to the definitive section and to identify the factors that could be associated with the appearance of false-negative cases. Secondarily, to evaluate the pathological results of cases submitted to completion axillary lymph node dissection (ALND) for positive SLN.

**Methods:**

We performed a five-year review of cases (2011–2015), including patients with early-stage breast cancer undergoing SLN biopsy, with frozen section evaluation and subsequent definitive pathological analysis. These results were compared to calculate the false-negative rate and the factors associated with it. The histopathological findings were also evaluated in patients submitted to completion ALND.

**Results:**

A total of 281 patients were evaluated, identifying 18 cases with frozen section results as false negative (false-negative rate: 23.7%), and 55.5% of these cases were micrometastases. The false-negative rate in SLN with macrometastasis was 13.1% and for micrometastasis cases was 66.7% (*p* < 0.001). True-positive patients that were submitted to completion ALND had additional axillary lymph nodes with metastases in 28% of cases, whereas the group of false negatives had additional positive axillary lymph nodes in 40% of patients (*p* = 0.62).

**Conclusion:**

Frozen section analysis had a false-negative rate acceptable in SLN biopsy in our institution, and the micrometastasis in the SLN was the most important factor associated with the appearance of this phenomenon.

## Introduction

Axillary lymph node status is one of the most important prognostic factors in breast carcinoma. Axillary lymph node dissection (ALND) had been the gold standard method to adequately determine the axillary stage in these patients. However, at the beginning of the 1990s, sentinel lymph node (SNL) biopsy was introduced as an alternative to ALND in patients with early stage disease [1, 2]. Currently, SNL biopsy is well established as standard of care for patients with clinically node-negative invasive breast cancer with similar oncological results compared to ALND, and lesser morbidity [3, 4].

Completion ALND is usually recommended in cases with histologically positive SLN biopsy. Intraoperative assessment of SLN by frozen section could identify about 60% of positive SLN, allowing an immediate ALND and avoiding reoperation. However, only 20% of these frozen section procedures are positive for metastases [5]. Also, frozen section positive patients who had immediate ALND were not consistently spared a reoperation because more than half required further surgery for positive margins after attempted breast-conserving surgery [6]. In addition, it is well known that lymph node metastasis is more likely to be missed during the frozen section than on the definitive section [7].

The prospective randomised ACOSOG Z0011 trial (of ALND vs. no further surgery for SLN-positive patients) have reported no differences in the rate of local or distant relapse between patients who had ALND and those who did not [8, 9]. Although most patients in that trial had favourable clinical–pathological factors, the routine use of frozen section for the evaluation of SLNB is questionable [10].

We performed a five-year retrospective review of SLN biopsy specimens with intraoperative evaluation via frozen section to evaluate sensitivity, specificity, positive- and negative-predictive values, reasons for discordance between the frozen section and final diagnoses, and additionally, we evaluated the histopathological findings in patients who underwent axillary dissection by a positive SLN biopsy.

## Methods

Data were retrospectively collected from patients who underwent SLN biopsy and evaluated with frozen section and definitive section at Luis Razetti Oncology Institute in Caracas, Venezuela, between January 2011 and December 2015. Only patients with invasive breast carcinoma and clinically negative axilla were included, and those with ductal carcinoma *in situ* or prior neo-adjuvant chemotherapy were excluded from this study. Also, we excluded patients with unidentified SLN performed. Patients diagnosed as having positive SLNs based on the frozen section diagnosis immediately underwent ALND per the surgeon criteria.

Blue dye technique was performed for SLN biopsy procedure. It was injected periareolarly in an intradermal plane for localisation and identification of SLN. Intraoperatively, SLNs were identified by their blue colour in addition to those found suspicious on palpation and those with afferent lymphatic vessels stained blue. ALND was performed if no SLN could be identified.

In all cases, the excised SLNs were submitted for immediate frozen section diagnosis. First, a careful gross examination, dissection of adipose tissue, and several sampling cuts of tissues were done [11]. Smaller nodes were sliced in two, while bigger nodes were sliced each 3–5 mm. Afterwards, sampling tissues were processed by freezing them with frozen aerosol sprays and put onto the cryostat for sectioning (the temperature inside was about −20° to −30° C). The tissue sections are cut and picked up on glass slides, which are then ready for staining with a modified haematoxylin and eosin (H&E) stains. Thereafter, an experienced pathologist evaluated these slides under the microscope and arrived at a diagnosis. Most of these cases were examined with touch imprint method too. The remaining tissues were sectioned at 2 mm intervals, fixed in formalin, embedded in paraffin, and entirely submitted for definitive diagnosis.

The results of frozen section and definitive section were compared regarding the pathological diagnosis for the SLNs. The standard was based on the results for the definitive section. In addition, we compiled clinicopathological data including patient age, histologic tumour type, tumour size, and metastasis size as well as follow-up data including subsequent ALND and chemoradiation treatment. Micrometastases were defined as a metastatic focus greater than 0.2 mm but less than or equal to 2.0 mm.

On the other hand, we determined either the total number of axillary lymph nodes (no-SLNs) in those patients who underwent ALND and the proportion of those with metastases. Data were entered on SPSS version 22 and sensitivity, accuracy, negative-predictive value, and false-negative rate were calculated from 2 × 2 tables. False-negative cases were defined as patients in whom a negative frozen section result was obtained, and whose final permanent paraffin section was positive. The chi-square test or the Fisher’s exact test was used for the comparison of the categorical variables. Logistic regression models were fitted to estimate the odds ratio with a 95% CIs with two-sided *p* values to assess the association of several variables with a false-negative diagnosis of frozen section of SLN. A *p* value < 0.05 was considered statistically significant.

## Results

A total of 281 patients were included and 620 SLNs were examined between January 2011 and December 2015. The median of SLNs dissected per patient was 2 (range, 1–7). The age of the patients ranged between 24 and 90 years (median, 54 years). The mean primary tumour size in the surgical specimen of the breast was 2.3 cm (SD ± 1.21). Most patients were in the pT2 category with 134 patients (47.7%), following by pT1c with 93 patients (33.1%). Two cases (0.7%) were occult breast carcinoma. The predominant histological type was invasive ductal carcinoma with 224 cases (79.7%). The clinicopathological data of the patients are summarised in Table 1.

A positive SLN biopsy was found in 76 of 281 (27.04%) patients in the definitive histological evaluation. Overall, frozen section had a sensitivity of 76.30%, a specificity of 99.02%, and a diagnostic accuracy of 92.88%. The positive- and negative-predictive value were 96.7% and 91.9%, respectively. Table 2 shows these results.

In 18 patients with positive nodes (6.4%), frozen section was negative, but the final examination on paraffin section gave a positive result (false negative rate, 23.7%). Of these, 10 cases (55.6%) were micrometastases.

Frozen section analysis could determine in seven of 281 patients the presence of micro-metastasis (2.5%); however, two of them were misinterpreted as positive because the definitive diagnosis was histiocytes even in a second review of the frozen section (false-positive rate: 0.71%).

False-negative rate was 66.7% and 13.1% in micrometastatic and macrometastatic disease, respectively (*p* < 0.001). In the same way, we found in patients with more than three SLNs dissected, that the false-negative rate is higher than in those with a lesser quantity of SLNs recovered (*p* = 0.029). However, we did not find a statistically significant difference in the other variables included, tumour size (*p* = 0.680), lymphovascular invasion (*p* = 0.942), and multicentricity (*p* = 0.611). These findings are summarised in Table 3.

In multivariate logistic regression analysis, we identified that only the size of metastases was a variable predictor for false-negative diagnosis. Thus, the smaller the SLN metastases, higher the odds of a false-negative diagnosis, with an OR of 13.25 (95% CI: 3.59–8.90; *p* = 0.001). The other variables did not reach statistical significance in final fitted model. These results are shown in Table 4.

In the group of patients with positive SLN, the mean number of dissected SLNs was 2.28 (SD ± 1.22). We also found that the number of the positive SLN recovered was greater in the group of true-positive case compared with false-negative case (*p* = 0.037) (uncharted results).

In true-positive patients, completion ALND was performed in 57 of 58 patients (98.28%), and it was more common than in the false-negative case with 5 of 18 patients (27.8%), (*p* < 0.001). We also analysed the status of the rest of axillary lymph nodes regarding if they were harbouring metastases or not. Thus, we found that 16 of 57 (28%) true-positive patient that were submitted to completion ALND had additional axillary lymph nodes with metastases. On the other hand, the group of false negatives had additional positive axillary lymph nodes in two of five patients (40%) (*p* = 0.62) (Table 5).

Patients who were taken to ALND for positive SLN biopsy had a median of dissected axillary lymph nodes of 14.00 (SD ± 4.15). Likewise, the median of positive axillary lymph nodes with metastases in such ALND was 2.83 (SD ± 1.85) (uncharted results).

## Discussion

In this study, we determined that the discordance between frozen section analysis and definitive diagnosis of SLN biopsy in patients with breast cancer occurred in 23.7%. Previous studies have reported false-negative rates that have ranged from approximately 10% to 60%; however, most of these were between 15% and 20% [12–17]. Our pathologists performed a frozen section evaluation of the sentinel node with one or two slices when the sentinel node was small. On the other hand, the larger nodes were evaluated by slices every 3 or 4 mm. There is a possibility that our pathologists had considered some nodes as small and made only one slice, when they should have made more than one cut. This argument could explain why the false-negative rate observed in our study is somewhat higher than the average.

As expected, the size of the metastasis in the SLN was associated with a false-negative diagnosis in the frozen section analysis. It was demonstrated in the univariate logistic regression analysis, where micrometastasis in the SLN had an odds ratio of 13.25. In the study done by Wong et al. [14], they reported a false-negative rate in patients with micrometastasis of 59.8%, as high as we found in our study (66.7%). In the same way, Holck et al. observed that micrometastasis in SLN was presented in 21 of 28 patients with false-negative result in frozen section analysis [12].

Completion ALND in patients with early-stage breast cancer has been recently questioned. In regard with this, there are two landmark trials, the IBCSG study 23-01 [18] and ACOSOG Z0011 trial [8]. Accordingly, the repercussion and true relevance of micrometastasis in the SLN may still be a topic of debate; however, the use of frozen section analysis for SLN biopsy could be useful since it is possible to perform the ALND in the same surgical event, saving costs.

In our study, patients with SLN with macrometastasis submitted to frozen section analysis had a false-negative rate of 13.1%. Only 27.8% of these cases were taken to ALND based on medical criteria. Thereby, only 11.1% of those had additional metastatic axillary lymph nodes. Similarly, Polig et al. [15] reported that only 15% of the false-negative patient had other nodes positive in ALND, a fact that supports the concept that most these patients would present limited disease.

In the literature, most of the studies have reported a true-positive rate in SLN biopsy with frozen section around 25% and this could save an additional surgery. However, there is an important trend towards diminishing of SLN frozen section evaluation, and this was observed in a study with 7509 patients over a 10-year period published by Weber et al. [10]. They showed declining rates of intraoperative frozen section from 100% to 62% (*p* < 0.001) and, for patients in whom frozen section was either negative or not done, a declining rate of completion ALND in SLN-positive patient. Also, they determined that if the Z0011 criteria had been applied in their cohort, 66% (4159 of 6327) of all frozen section and 48% (939 of 1953) of all ALND would have been eliminated, sparing 13% of their total patient population the morbidity of ALND.

## Conclusion

Several potential limitations to this study included the small number of patients, and some data were missed due to the retrospective review. We believe that comprehensive information of the definitive histopathological findings either of the primary tumour and SLNs may help in making a better decision to completion ALND without an intraoperative frozen section analysis.

## Competing interests

The authors declare that they have no conflict of interests.

## Figures and Tables

**Table 1.** Clinicopathological characteristics of patients.

Characteristics	N° (%)
Patients (n)	281 (100)
Age (years): median (range)	54 (24–90)
Tumour size (pT) pT0 pT1 ( ≤ 2 cms) pT1a ( > 1 mm, ≤ 5 mm) pT1b ( > 5 mm, ≤ 10 mm) pT1c ( > 10 mm, ≤ 20 mm) pT2 ( > 2 cm, ≤ 5 cm) pT3 ( > 5 cm)	2 (0,7) 136 (48,4) 14 (5,0) 29 (10,3) 93 (33,1) 134 (47,7) 9 (3,2)
Nodal status (pN) pN0 pN1 pN1mi pN2	203 (72,2) 69 (24,6) 15 (5,3) 9 (3,2)
AJCC 7°ed staging IA IB IIA IIB IIIA	100 (35,6) 6 (2,1) 125 (44,5) 40 (14,2) 10 (3,6)
Histological grade 1 2 3	52 (18,5) 185 (65,8) 44 (15,7)
Histological type Ductal Lobulillar Ducto-lobulillar Papilar Mucinous Others	224 (79,7) 18 (6,4) 12 (4,3) 13 (4,6) 5 (1,8) 9 (3,2)
Lymphovascular involvement No Yes	167 (59,4) 114 (40,6)
Multicentricity No Yes	252 (89,7) 29 (10,3)
Type of surgery PM + SLNB TSM + SLNB PM + SLNB + ALND TSM + SLNB + ALND	166 (59,1) 55 (19,6) 39 (13,9) 21 (7,5)
Condition of the rest of the axilla No ALND ALND without nodes Mt. ALND with nodes Mt.	211 (75,1) 50 (17,8) 20 (7,1)

MPO: oncologic partial mastectomy, SLNB: sentinel lymph node biopsy, TSM: total simple mastectomy, ALND: axillary lymph node dissection, Mt: metastatic

**Table 2.** Comparison of frozen section versus definitive section of sentinel lymph node biopsy.

Frozen section	Definitive section		
	Positive	Negative	Total
Positive	58	2	60
Negative	18	203	221
Total	76	205	281
Sensitivity %			76.30
Specificity%			99.02
False-negative rate %			23.70
Positive prediction rate %			96.70
Negative prediction rate %			91.90
Diagnostic accuracy %			92.88

**Table 3.** False-negative rate and sensitivity of frozen section of sentinel lymph node by related pathological factors.

Variables	N° (%)	TP	FN	Se %	FNR %	*p* Value
Total	281 (100)	58	18	76.3	23.7	–
**SLN metastasis size**						
Micrometastasis	15 (19.7)	5	10	33.3	66.7	**< 0.001**
Macrometastasis	61 (80.3)	53	8	86.9	13.1	

**Number of SLNs dissected**						
≤ 2	50 (65.8)	42	8	84.0	16.0	**0.029**
> 2	26 (34.2)	16	10	61.5	38.5	

**Tumour size**						
≤ 2 cm	37 (48.7)	29	8	78.4	21.6	0.680
> 2 cm	39 (51.3)	29	10	74.4	25.6	

**Lymphovascular involvement**						
Absent	29 (38.2)	22	7	75.9	24.1	0.942
Present	47 (61.8)	36	11	76.6	23.4	
**Multicentricity**						
Absent	68 (89.5)	52	16	76.5	23.5	0.926
Present	8 (10.5)	6	2	75.0	25.0	

Bold values represent statistical significance. TP: true positives, FN: false negatives, Se: sensitivity, FNR: false-negative rate, SLN: sentinel lymph node

**Table 4.** Multivariate logistic regression analysis of false-negative rate of SLNs.

	*N*° (%)	TP	FN	TFN (%)	OR, (IC 95%)	*p* Value
Total	281 (100)	58	18	23.7	–	–
**SLN metastasis size**						
Micrometastasis	15 (19.7)	5	10	66.7	13.25 (3.6–48.9)	** < 0.001**
Macrometastasis	61 (80.3)	53	8	13.1	1	

**Number of SLNs dissected**						
> 2	26 (34.2)	16	10	38.5	3.28 (1.1–9.8)	0.373
≤ 2	50 (65.8)	42	8	16.0	1	

**Tumour Size**						
> 2 cm	39 (51,3)	29	10	25.6	1.25 (0.4–3.6)	0.508
≤ 2 cm	37 (48,7)	29	8	21.6	1	

**Lymphovascular involvement**						
Absent	29 (38.2)	22	7	24.1	1.04 (0.4–3.1)	0.503
Present	47 (61.8)	36	11	23.4	1	

**Multicentricity**						
Present	8 (10.5)	6	2	25.0	1.08 (0.2–5.9)	0.563
Absent	68 (89.5)	52	16	23.5	1	

Bold values represent statistical significance. TP: true positives, FN: false negative, Se: sensitivity, FNR: false negative rate, SLN: sentinel lymph node, OR: odds ratio.

**Table 5.** Analysis of patients with positive SLN submitted to completion ALND.

Completion ALND			
**Performed**			**Not performed**
**Neg ALNs**		**Pos ALNs**	
**True positives**	41	16	1
***n* (%)**	(70.7)	(27.6)	(1.7)

**False negatives**	3	2	13
***n* (%)**	(16.7)	(11.1)	(72.2)

SLN: sentinel lymph node, ALND: axillary lymph node dissection, ALN: axillary lymph nodes
